# Fabrication and Dielectric Characterization of Stable Oil in Gelatin Breast Tissue Phantoms for Microwave Biomedical Imaging

**DOI:** 10.3390/mi16101189

**Published:** 2025-10-21

**Authors:** Héctor López-Calderón, Víctor Velázquez-Martínez, Celia Calderón-Ramón, Juan Rodrigo Laguna-Camacho, Benoit Roger-Fouconnier, Jaime Martínez-Castillo, Enrique López-Calderón, Javier Calderón-Sánchez, Jorge Chagoya-Ramírez, Armando Aguilar-Meléndez

**Affiliations:** 1 Facultad de Biología, Universidad Veracruzana, Xalapa 91090, Mexico; hectolopez@uv.mx; 2 Facultad de Ingeniería Mecánica y Eléctrica, Universidad Veracruzana, Poza Rica 93390, Mexico; ccalderon@uv.mx (C.C.-R.); jlaguna@uv.mx (J.R.L.-C.); enriquelopez@uv.mx (E.L.-C.); jacalderon@uv.mx (J.C.-S.); jchagoya@uv.mx (J.C.-R.); 3 Facultad de Ciencias Químicas, Universidad Veracruzana, Coatzacoalcos 96538, Mexico; broger@uv.mx; 4 Centro de Investigación de Micro y Nanotecnología, Universidad Veracruzana, Boca del Río 94294, Mexico; jaimartinez@uv.mx; 5 Facultad de Ingeniería Civil, Universidad Veracruzana, Poza Rica 93390, Mexico; armaguilar@uv.mx

**Keywords:** dielectric characterization, microwave biomedical imaging, tissue-mimicking materials, biofabrication

## Abstract

Breast tissue-mimicking phantoms are essential tools for validating microwave imaging systems designed for early breast cancer detection. In this work, we report the fabrication and comprehensive characterization of oil-in-gelatin phantoms emulating normal, benign, and malignant breast tissues. The phantoms were manufactured with controlled mixtures of kerosene, safflower oil, and gelatin, and their dielectric properties were experimentally evaluated using a free-space transmission method with a Vector Network Analyzer across the 100 MHz–10 GHz range. Results demonstrated significant contrast in permittivity and conductivity among the different tissue types, consistent with values reported in the literature. Long-term stability was confirmed for up to six months under controlled storage. Additional structural and thermal characterization was performed using Fourier transform infrared spectroscopy (FTIR), differential scanning calorimetry (DSC), and thermogravimetric analysis (TGA), providing insight into molecular composition and thermal response. The proposed method enables reproducible, low-cost, and stable phantom fabrication, offering reliable tissue models to support experimental validation and optimization of microwave-based breast cancer detection systems.

## 1. Introduction

The probability of developing invasive breast cancer during a woman’s lifetime has been increasing, despite early detection campaigns for this type of cancer. Breast cancer is one of the most common cancers in women, and statistics indicate that in recent decades, more women are dying from this disease; even worse, these rates have been increasing in young women. Therefore, it is in our interest to participate in the construction of a prototype that operates at frequencies different from those used in X-ray mammography, as it is intended for use only in women over 40 years old. Microwave technology has been studied in recent decades as a viable alternative to achieve this objective, due to the high contrast produced in breast tissue, that is, between normal or healthy tissue and malignant tissue in this frequency range [1]. To perform tests on phantoms, it is required to model the breast tissue as close as possible to a real breast, which is the focus of this work. The classification of breast tissue is generally considered in three parts: the first is adipose or fatty tissue, considered normal tissue; cysts and fibroadenomas are classified as benign tissue; and invasive and non-invasive lobular and ductal carcinoma are classified as malignant tissue [2]. The high dielectric contrast between normal and malignant breast tissue in the microwave frequency range has enabled ongoing research over the last few decades to apply microwave imaging technology for the early detection of breast cancer [1,2,3]. The diagnosis of breast cancer with this technology is promising; prototypes have been made experimentally, supported by many investigations that have provided significant results. It is crucial for us to verify that the phantoms are correctly elaborated. Several studies have modeled breast tissue using phantoms that emulate dielectric parameters [4,5], some of which have already progressed to clinical evaluation. To achieve a good response in terms of image resolution, some researchers have used 3D morphology as the material for the phantoms in the construction of their experimental prototypes [6].

Depending on the base ingredient used to make them, various types of phantoms can be sensitive to environmental exposure and have a limited shelf life. In addition, it is desirable that a phantom be manufactured economically, repeatable, with adequate dielectric properties, as stable as possible over time, and that it can be tested flexibly but reliably in a variety of scenarios [7].

On the one hand, significant changes in dielectric properties have been observed depending on the mixture of ingredients used, which has been a problem. Some studies report stability of the phantoms over a maximum period of six months. On the other hand, breast tissue modeled using heterogeneous mixtures of oil-in-gelatin with various oil percentages demonstrated long-term stability in the range of 1–6 GHz [8]. Skin, adipose tissue, fibroglandular tissue, and tumor were simulated with mixtures of TX-151 polyethylene powder and water in different percentages by [9] in the range of 3 to 10 GHz. Due to their physical characteristics, the modeling of various types of malignant tissue has been performed with different types of spherical, hemispherical, ellipsoidal, or spiculated phantoms [10], which correspond to the variety of tumor shapes. The use of polyurethane as a base ingredient, whose dielectric properties are controlled by adding carbon black and graphite to the rubber mixture, has also been reported, resulting in robust, easy-to-use, and representative phantoms with accurate dielectric properties [11].

Oil-in-gel mixtures have yielded good results; however, they exhibit characteristics that make them highly sensitive to environmental exposure and have a limited shelf life, which contrasts with their cost and stability during the first few months after manufacture. In [12], reported results using materials modeling healthy and malignant tissues, which allowed for the construction of breast phantoms, using various mixtures of kerosene, oil in gelatin, and formaldehyde, among others, to simulate the dielectric properties of these phantoms from 500 MHz to 20 GHz. The materials obtained were solid, elastic, and stable for a considerable period. Multimodal breast phantoms [13] have been developed and utilized in electromagnetic-based imaging modalities, including microwave, ultrasound, mammography, magnetic resonance imaging, and computed tomography. In [14], the development of phantoms in the frequency range of 6–14 GHz is reported, using diffracted field reconstruction. Several investigations have proposed the use of phantoms, reporting that the dielectric properties of different mammary tissues can be reproduced by varying the proportions of their ingredients. These proportions exhibit characteristics of stability over longer periods, compared to oil-in-gelatin mixtures [15]. Another study by [4] used phantoms in the frequency range of 2–14 GHz. In [16], four phantoms were fabricated with cylindrical inclusions to frequency ranges of 1.5–10 GHz.

The work presented in this article focuses on the development and characterization of phantoms representing normal, malignant, and benign breast tissue, which enable the emulation and modeling of the dielectric properties of biological breast tissue. These properties are key to the system or prototype to be implemented in the future. An analysis of the procedures used to produce the phantoms was conducted, considering the materials for their manufacture, to ensure they were of the same size and geometric shape, utilizing a hemispherical mold. According to some research, various types of phantoms have been developed for the fabrication of healthy, malignant, and benign breast tissue, as shown in Table 1.

Dielectric properties of permittivity and conductivity of each phantom were obtained using Keysight Materials Measurement Suite 2020 software from the Vector Network Analyzer (VNA) measurements. A free-space transmission method was used experimentally, connecting two Vivaldi antennas to the VNA, with one antenna functioning as a transmitter and the other as a receiver [18,19,20]. Measurements were performed over a frequency range of 100 MHz to 10 GHz, obtaining transmission and reflection coefficients of each measured phantom [29].

In this context, the present study focused on the fabrication and characterization of breast tissue phantoms representing normal, benign, and malignant tissues through reproducible oil-in-gelatin formulations. The mixtures were optimized to achieve dielectric properties consistent with those of biological tissues, while maintaining comparable size and geometry across all phantoms to facilitate reliable measurements and future integration into experimental prototypes.

The novelty of this work lies in the development of tissue-mimicking materials that are not only low-cost and reproducible but also prepared from reagents that are relatively easy to obtain and handle. Unlike many previously reported gelatin-based biomimetic materials, the phantoms described here exhibit long-term stability for at least six months under optimal storage conditions, ensuring their suitability for extended experimental campaigns. This balance of affordability, accessibility, and durability constitutes the primary advantage of the proposed formulation, supporting its potential for advancing microwave-based breast cancer imaging research.

## 2. Materials and Methods

### 2.1. Breast Tissue Phantom Fabrication

The preparation of solutions and mixtures to obtain breast phantoms is primarily based on modifications to the methodology by Torrealba-Meléndez [20]. In Table 2, the quantities used for the preparation of the mixtures to construct the three tissue models are described. Briefly, a gelatin (gel strength 200, Knox, Oak Brook, IL, USA) solution (17% *w*/*v*) was prepared using double-distilled water as solvent. Likewise, a mixture of safflower oil (Oleico, San Luis Potosí, Mexico) and kerosene (Meyer, Mexico City, Mexico) was prepared in equal proportions (1:1). Both solutions were mixed in different proportions depending on the tissue: (a) Normal tissue with 80% oil and 20% gelatin; (b) Benign tumor tissue with 20% oil and 80% gelatin; and (c) Malignant tumor tissue with 10% oil and 90% gelatin. Once the solutions were obtained, they were placed in hemispherical molds for 5 days to allow the formaldehyde cross-linking process to take place.

**Figure 1:**
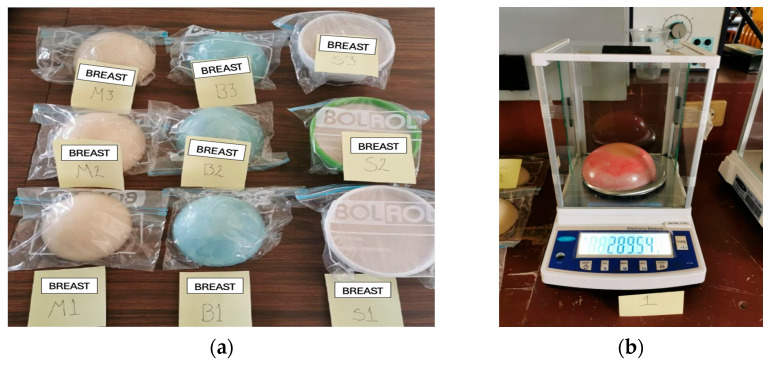
(**a**) Three phantoms were manufactured for each type of tissue: malignant (M), benign (B), and normal (S). (**b**) Weight of the phantoms using an analytical scale.

The phantoms elaborated are shown in Figure 1, with a base diameter of 10 cm and a height of 5 cm. To maintain representativeness and reliability in this study, three phantoms were manufactured for malignant tissue (M), three for benign tissue (B), and three for normal tissue (S). Each sample was manufactured with the same geometric shape using a plastic mold, and the weight of each phantom was controlled using an analytical scale, as shown in Figure 1.

The stability of the samples of each tissue type was evaluated six months after their manufacture. Visibly, we can assure that the properties of each one remained unchanged, since we took the precaution of storing the samples in a resealable plastic bag, which prevented their contact with the environment, as has been reported in the literature.

**Figure 2:**
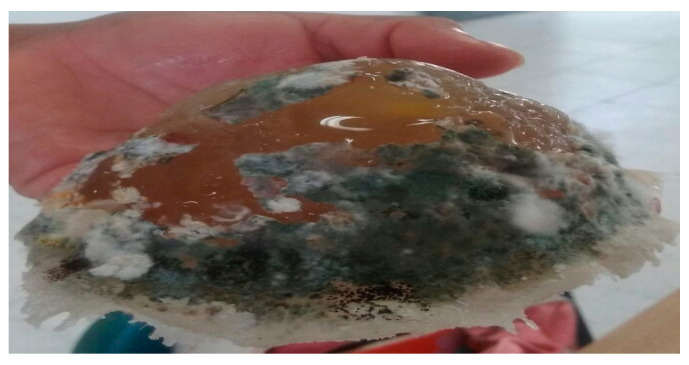
Decomposition of the normal tissue phantom after 2 months.

Only one sample was left out of these storage conditions for testing purposes, and its characteristics changed significantly, to the point that it presented mold at the end of the third week. Thus, it is proven that stability is a fact if they are properly preserved. Figure 2 shows the sample in a state of decomposition.

### 2.2. Characterization of the Phantoms

#### 2.2.1. Vector Network Analyzer (VNA)

In the present work, the VNA (Keysight, Santa Rosa, CA, USA) is used to measure breast tissue phantoms that have been developed to model both normal and malignant tissues. To perform these measurements, the Agilent 85070 kit (Keysight, Santa Rosa, CA, USA) and the Keysight Materials Measurement Suite 2020 software (v 20.0.24092501, Keysight, Santa Rosa, CA, USA) were used as an interface. This software controls the VNA and allows for measuring the transmission and reflection coefficients of the breast tissue phantoms, subsequently converting these coefficients into the complex permittivity of each phantom. It displays the measurement results in graphs. The complex permittivity is expressed by permittivity *ε*′ and imaginary electric permittivity *ε*″, which are related by Equation (1).

(1) $$\varepsilon =\varepsilon^{\prime }+j\varepsilon^{\prime \prime }$$

where *ε*′ is the electric permittivity in F/m, which represents the response of a material to an electric field, and *ε*″ is the imaginary electric permittivity and represents the loss factor of the material. The imaginary electric *ε*″ is expressed by the conductivity σ, which is related by Equation (2).

(2) $$\varepsilon^{\prime \prime }=\frac{\sigma }{\omega \varepsilon_{0}}$$

where *σ* represents the electrical conductivity in S/m, ω is the angular frequency, and $\varepsilon_{0}$ is the vacuum permittivity equivalent to 8.85 × 10^−12^ in F/m. By subtracting σ from Equation (2).

(3) $$\sigma =\omega \varepsilon^{\prime \prime }\varepsilon_{0}$$

**Figure 3:**
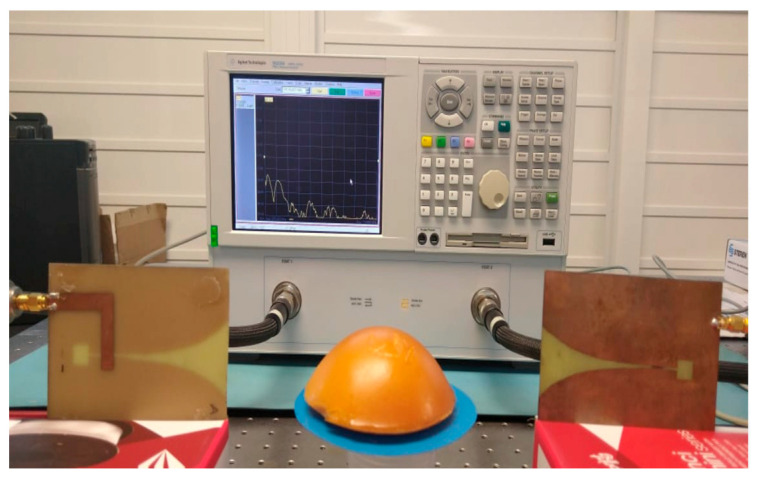
Measuring permittivity and conductivity with VNA.

Figure 3 shows one of the breast tissue phantoms, placed between two Vivaldi antennas that enable the use of the free-space technique between the VNA terminals.

This same configuration is used for each of the normal and malignant phantoms.

#### 2.2.2. Fourier Transform Infrared Spectroscopy (FT-IR)

FT-IR readings were taken to detect the characteristic signals of the compounds used to generate the different phantoms. The assays were carried out using a Nicolet iS10 instrument equipped with the Smart OMNI-Transmission accessory (Thermo Scientific, Waltham, MA, USA), taking readings in the range of 4000–650 cm^−1^ with a resolution of 4 cm^−1^. Essential FTIR^®^ software version 3.50.194 was used for the analysis of the spectra obtained.

#### 2.2.3. Differential Scanning Calorimetry (DSC)

The thermal properties of tissue phantom samples were determined using a DSC Q2000 calorimeter (TA Instruments, New Castle, DE, USA). Approximately 13 mg of tissue phantom samples were placed in hermetically sealed aluminum pans and inserted into the calorimeter head in a nitrogen environment, with a purge rate of 20 mL/min. The samples were then subjected to regular cooling from 20 °C to −50 °C at a rate of 5 °C/min. Then, all samples were regularly heated to 450 °C at the same scanning rate. Enthalpies and ice melting temperatures were analyzed using the manufacturer’s universal analysis software.

#### 2.2.4. Thermogravimetric Analysis (TGA)

Thermogravimetric analysis of tissue phantom samples was performed using a Q50 TGA equipment analyzer (TA Instruments, New Castle, DE, USA). Approximately 60 mg of the sample was placed on a platinum plate and introduced into the TGA furnace. The samples were heated from 40 °C to 600 °C at a rate of 5 °C/min under nitrogen gas. All weight loss events were analyzed and differentiated over time by using the TA universal analysis software (TRIOS v5.0, TA Instruments, New Castle, DE, USA).

## 3. Results

### 3.1. Vector Network Analyzer

**Figure 4:**
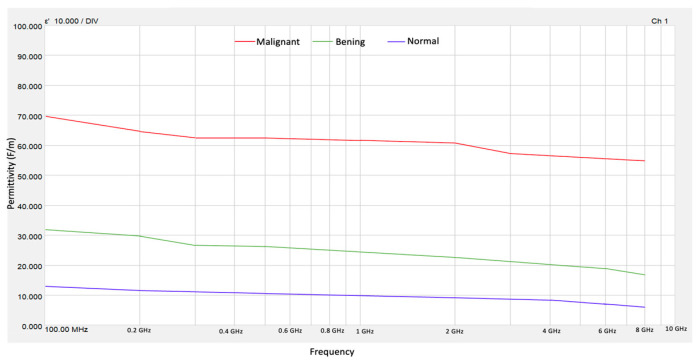
Electrical permittivity for normal, benign, and malignant tissue phantoms.

The results of the real electrical permittivity were obtained using Keysight Materials Measurement Suite 2020 software from the VNA measurements. Figure 4 shows the values obtained for *ε*′ of normal, benign, and malignant tissue phantoms. The high contrast in electrical permittivity between malignant and normal tissues occurs between frequencies of 0.4 and 8 GHz. This behavior does not occur in other frequency ranges, making the use of microwave technology to differentiate between malignant and healthy tissue a promising approach. On the other hand, the contrast between the permittivity of benign tissue and normal tissue is also evident in this frequency range. It has been considered that this distinction between these types of tissue cannot yet be taken for granted, so in this sense, only the high dielectric contrast between normal tissue and malignant tissue can be guaranteed.

**Figure 5:**
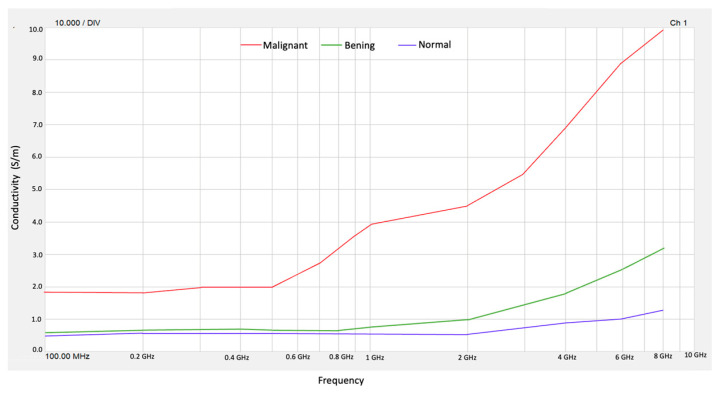
Conductivity for normal, benign, and malignant tissue phantoms.

Figure 5 shows the values obtained for the conductivity of normal, benign, and malignant tissue phantoms.

From the obtained values of *ε*″ in the frequency range from 100 MHz to 10 GHz, we obtained conductivity (*σ*) for normal tissue and malignant tissue phantoms. Then, the results obtained for permittivity *ε*′ and conductivity σ allow us to validate that benign, malignant, and normal breast tissue phantoms behave as reported values in the literature [1]. On the other hand, the high contrast in electrical conductivity between malignant tissue and normal tissue occurs at frequencies of 4 to 8 GHz. The contrast of the conductivity of normal tissue with respect to benign tissue is practically zero until 1 GHz, so it is not possible to distinguish between these types of breast tissue.

Table 3 presents the results obtained at the beginning of the experiment and after 6 months for each of the manufactured phantoms.

The values obtained in this study align with those from various projects carried out by other colleagues, including [13,20,30,31,32].

### 3.2. Fourier Transform Infrared Spectroscopy

**Figure 6:**
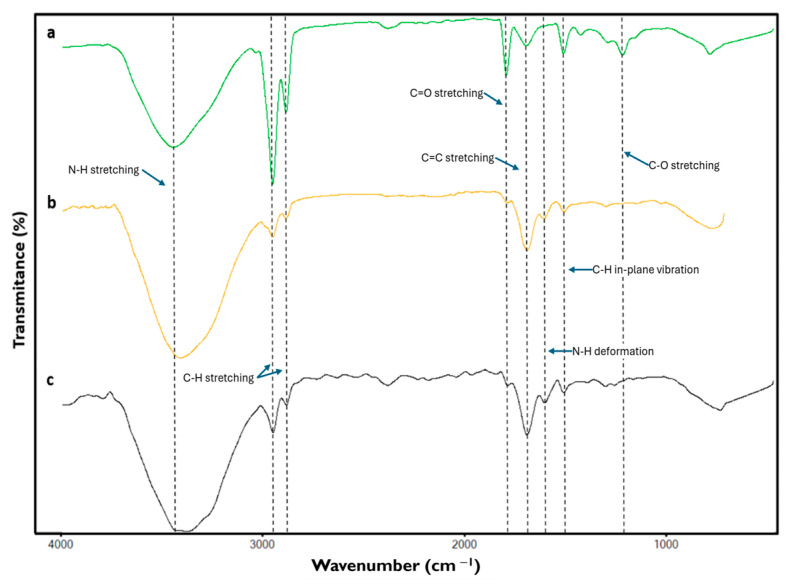
Fourier transform infrared spectroscopy spectra of the different phantoms emulating (**a**) normal, (**b**) benign, and (**c**) malignant breast tissue.

Among the signals yielded by the spectra of the different formulations of the phantoms, those bands corresponding to the presence of gelatin and the oils composing the samples are highlighted. Figure 6 shows the characteristic signals of all components present in the material.

The bands around 3426 cm^−1^, due to the stretching of the N-H bond of amide groups, can be attributed to the presence of gelatin [33,34,35]. At 2925 and 2857 cm^−1^ signals due to C-H stretching were found, resulting from the presence of hydrocarbon chains possibly of an aliphatic nature [34,36,37,38,39] mainly due to the composition of oils contained in the samples, likewise the band at 1747 cm^−1^ reflects the presence of C=O stretching of the carbonyl of the ester groups [36,37,39] attributable to the components contained in the oil solution of the phantoms.

The signal found at 1645 cm^−1^ corresponds to the stretching of the C=C belonging to amide I [34,38,40] of gelatin, a compound that also generates the band around 1555 cm^−1^ caused by the presence of the deformation of the N-H bonds of amide II [33,38].

In the case of the band at 1458 cm^−1^, it is a signal due to the C-H in-plane vibration, possibly resulting from the aliphatic chains present in the sample [34,38,41], which is attributed to the presence of oils. Finally, the band at 1160 cm^−1^ is attributed to C-O stretching of the glycolipids [36,39,42], which may reflect possible interactions between gelatin and oils.

Considering the signals detected in the spectra of the different phantoms, a relationship between the strength of the signals and the amount of the components used for the formulation of each sample can be observed.

It is noticeable that the signals corresponding to the presence of the oils were detected with greater strength in the normal tissue phantom sample, whereas they decreased in those samples representing benign and malignant tumor tissue, respectively. Similarly, the signals corresponding to the presence of gelatin in the samples were detected primarily in those representing malignant and benign tumor tissues, and their strength diminished when readings were performed on samples corresponding to normal tissue.

### 3.3. Differential Scanning Calorimetry

**Figure 7:**
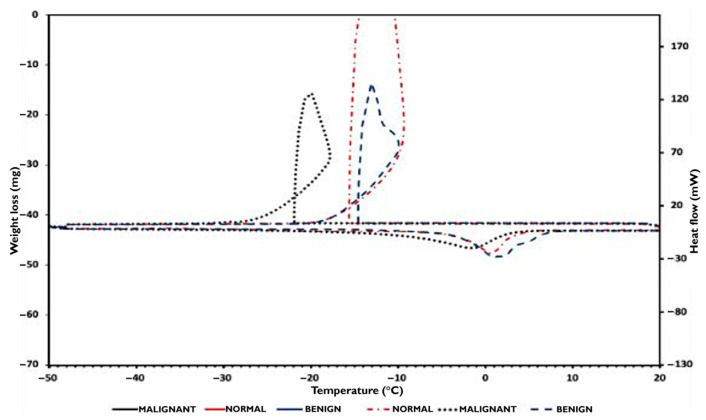
DSC cooling and heating sample phantom’s curves.

In Figure 7, the DSC cooling and heating sample curves were plotted between −50 °C and 20 °C. During cooling, each DSC trace is characterized by the presence of a sharp exothermic peak that occurred at −14.5 °C, −15.5 °C, and −22 °C for benign, normal, and malignant samples, respectively. These peaks are attributed to the crystallization of water contained in the samples. As the tissue phantoms were prepared from gelatin solutions, both free and bound water are present in the samples, allowing for the easy detection of free water crystallization by DSC during sample cooling.

During the samples’ normal state, the ice melting signals are shifted towards temperatures lower than 0 °C, and especially at −3 °C for both normal and benign samples, and at −10 °C for the malignant sample. These signals are characteristic of progressive ice melting in equilibrium with a remaining solution containing polymer, n-propanol, detergent, and benzoic acid, and correspond to the temperature at which the endothermic signal reaches its maximum. Using Equation (1), the mass of crystallizable and non-crystallizable water contained in the samples was determined and reported in Table 4.

### 3.4. Thermogravimetric Analysis

**Figure 8:**
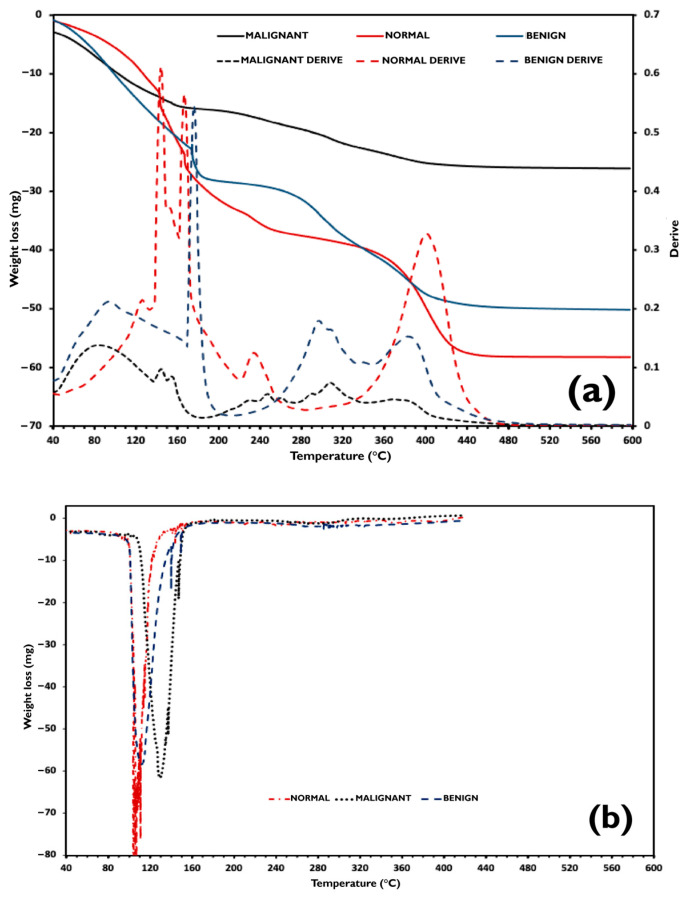
(**a**) TGA curves of the phantoms and their first derivative. (**b**) Comparison to the DSC heating curves plotted.

In Figure 8a, TGA traces and their first derivative curves are exhibited and compared to the DSC heating curves plotted in Figure 8b between 40 °C and 450 °C. As can be observed, in all TGA runs, the samples lose weight since they start the heating from 40 °C. Additionally, DSC endothermic signals detected at around 100 °C correspond to water evaporation, except for the malignant sample, which occurred at 105 °C. Consequently, in the first decomposition range between 40 °C and 170 °C, the TGA events correspond to the evaporation of crystallizable and non-crystallizable water, n-propanol, and benzoic acid contained in the samples. The subsequent TGA events detected between 170 °C and 650 °C are attributed to the decomposition of the hydrophilic polymers and the oil phase, which is composed of safflower oil and kerosene, decomposing and evaporating in the 250–600 °C and 177–343 °C temperature intervals, respectively [43,44].

In this study, it is appropriate to consider water in the tissue phantoms as either crystallizable or non-crystallizable water, rather than unbound and bound water. The samples were formulated with the same water-to-dry gelatin ratio and thus have the same maximum water weight fraction, which equals 0.848. However, the non-crystallizable water content in each sample differs from that in the others. For the normal sample, the amount of crystallized water measured by the DSC technique corresponds to the total quantity of water contained in the sample formulation. Non-crystallizable water was therefore detected, and thus, from Equation (4), it can be deduced that for this sample, Ccr almost equals Cw (0.848).

(4) Cnc=Cw−Ccr

where Cnc is the non-crystallizable water weight fraction, Cw the water weight fraction, and Ccr the crystallizable water weight fraction.

However, as the benign and malignant samples contain excess water, Cw had to be experimentally determined. On the other hand, as reported by [45] in these gelatin–water systems, the endothermic ice melting peaks are due to the melting of ice in equilibrium with a solution of gelatin whose concentration varies as it is continuously diluted during the ice melting process.

Indeed, as water crystallized in all the tissue phantom samples, this signifies that all samples contained water above the critical water concentration C*. Thus, when water crystallization occurred in the samples, water and salt remained in the amorphous phase at a concentration C*, and the malign sample presented a higher degree of supercooling (i.e., 22 °C). During the heating of the samples, the ice melting occurred at T*, and ice progressively melts during heating, diluting the remaining solution until it reaches Cw at temperature Tw. The higher ΔT = Tw − T, higher ΔC = Cw − C*, and thus, it can be deduced that C* malign is much smaller than C* of normal and benign breast tissue phantoms.

### 3.5. Composition and Non-Crystallizable Water

FTIR results show that, as the oil content increases in the phantoms, normal > benign > malignant, the aliphatic CH bands (≈2925/2857 cm^−1^) and the ester carbonyl band (≈1747 cm^−1^) become more intense, whereas with higher gelatin content, the amide I and II bands are reinforced. This behavior indicates a more protein-rich matrix in the tumor phantoms and a more lipid-rich matrix in the normal one.

In DSC, this higher protein fraction translates into a greater proportion of water associated with the network, 0% for normal, 21% for benign, and 33% for malignant. The ability of gelatin to retain water in confined domains explains the increase in non-crystallizable water as the gelatin fraction, as detected by FTIR, increases.

### 3.6. Gelatin–Lipid Interactions and Thermal Shifts

The band at ≈1160 cm^−1^ suggests gelatin–oil interactions; when gelatin predominates, these interactions, along with a higher density of hydrogen bonds, shift the water phase transition events. The malignant phantom crystallizes in a more “supercooled” state and melts at lower temperatures compared to the normal/benign ones. FTIR supports the greater rigidity of the network, showing more intense amide bands, while DSC expresses this behavior as increased supercooling and depressed melting temperatures.

## 4. Conclusions

Oil-in-gelatin breast tissue phantoms developed in this study successfully reproduced the dielectric behavior of normal, benign, and malignant tissues within the 0.1–10 GHz frequency range. The measured permittivity and conductivity values agreed with those reported in the literature for biological tissues, confirming the reliability of the fabrication method.

The combined FTIR, DSC, and TGA analyses revealed consistent correlations between molecular composition and thermal behavior. Higher gelatin content increased amide signals, non-crystallizable water, and supercooling; while higher oil content enhanced aliphatic and ester bands. These findings demonstrate that the oil-gelatin ratio governs both dielectric and structural properties of the phantoms.

Compared to previously reported gelatin-based formulations, the proposed materials are low-cost, reproducible, and stable for at least six months under controlled storage conditions, representing a practical advantage for long-term experimental validation in microwave breast imaging systems.

## Figures and Tables

**Table 1:** Summary of formulations and materials reported in the literature for breast tissue phantom fabrication.

Reference	Formulation
[6]	Distilled water, Triton X-100, and salt
[11]	Polyurethane, graphite powder, and carbon black
[14]	Oil, PCB, and silicone. ABS, gelatin, and water
[16]	Vaseline, wheat flour, and gelatin
[17]	Urethane coating with 25% graphite, canola oil, glycerin, and water
[18]	ABS, gelatin, and water
[19]	Gelatin, surfactant, formaldehyde, and oil
[20]	Oil, kerosene, gelatin, glycerin, distilled water, and formaldehyde
[21]	Sunflower oil, water, and gelatin
[22]	Distilled water, sugar, agar, and salt in various proportions
[23]	Bean oil, corn flour, and water
[24]	Gelatin
[25]	Styrene–Acrylonitrile
[26]	Agar, agarose, and polyvinyl alcohol
[27]	Agarose, double-distilled degassed water, ethanol, and sodium chloride
[28]	Mix of oils, water, and agar

**Table 2:** Components and their quantity used for each tissue phantom formulation.

Component	Normal	Benign	Malignant
Gelatin (% *p*/*v*)	03.24	11.79	13.27
Oil (% *v*/*v*)	74.78	18.49	08.52
Formaldehyde * (% *v*/*v*)	00.08	00.27	00.31
Liquid detergent (% *v*/*v*)	03.04	00.70	00.45
n-propanol * (% *v*/*v*)	00.76	02.77	03.12
Water (% *v*/*v*)	18.08	65.91	74.15
Benzoic acid * (% *v*/*v*)	00.02	00.07	00.08

* Reagents were purchased from Meyer, CDMX, Mexico. Detergent was from P&G, Cincinnati, OH, USA.

**Table 3:** Permittivity and conductivity values at the beginning and after 6 months.

Phantom	Beginning			At 6 Months		
	Permittivity (F/m)			Permittivity (F/m)		
	2 GHz	4 GHz	6 GHz	2 GHz	4 GHz	6 GHz
Normal	9	8	7	8.8	7.9	6.9
Benign	23	20	18	22.8	20	18
Malignant	58	55	55	57	55	55
	Conductivity			Conductivity		
Normal	0.5	0.9	1	0.5	0.9	1
Benign	1	1.8	2.5	0.9	1.7	2.4
Malignant	4.5	7	9	4.5	7	8.9

**Table 4:** Water contained in the TMM.

Watter (wt%)	Normal	Benign	Malignant
Crystallizable	28.53	47.43	43.25
Non-crystallizable	0	21.21	32.75

## Data Availability

The original contributions presented in this study are included in the article. Further inquiries can be directed to the corresponding author.
